# Effects of Supplementation of Boric Acid in Dietary and Drinking Water on Performance, Incubation Characteristic and Intestinal Microbiota in Different Quail Varieties (*Coturnix coturnix japonica*)

**DOI:** 10.1002/vms3.70513

**Published:** 2025-07-28

**Authors:** Sultan Aslan, Ülkü Gülcihan Simsek, Mehmet Eroğlu, Seda İflazoğlu Mutlu

**Affiliations:** ^1^ Department of Animal Science, Faculty of Veterinary Medicine Dicle University Diyarbakır Türkiye; ^2^ Department of Animal Science, Faculty of Veterinary Medicine Firat University Elazığ Türkiye; ^3^ Department of Animal Science, Faculty of Veterinary Medicine Siirt University Siirt Türkiye; ^4^ Department of Animal Nutrition and Nutritional Diseases, Faculty of Veterinary Medicine Firat University Elazığ Türkiye

**Keywords:** boric acid | gut health | hatchability | laying performance | quail | variety

## Abstract

**Background:**

Boric acid is an important alternative to antibiotics due to its positive effects on the immune system, endocrine system, lipid metabolism, mineral metabolism and energy metabolism. Different doses of boric acid supplemented to diet and water may influence performance, hatchability and intestinal bacterial load in Japanese quails with various feather colours.

**Objective:**

The aim of the study was to determine the effects of different doses of boric acid, supplemented in feed and water, on performance, hatchability and intestinal bacterial load in Japanese quails with different feather colours.

**Methods:**

A completely randomized 5 × 4 factorial design was implemented, consisting of five dietary treatments and four feather colour groups. The dietary treatments were control (basal diet without supplementation), F100 (100 mg/kg boric acid added to feed), F300 (300 mg/kg boric acid in feed), W100 (100 mg/L boric acid added to drinking water) and W300 (300 mg/L boric acid in water). A total of 300 Japanese quails (*Coturnix coturnix japonica*) with different feather colour genotypes—yellow (Y), white (W), grey (G) and black (B)—served as the experimental subjects. Each treatment group included 60 quails, with 15 individuals from each feather colour and experiment lasted for 90 days.

**Results:**

Supplementation of boric acid to feed and water did not affect egg production, feed consumption, feed conversion rate, viability or water consumption (*p* > 0.05). In the F300 group, hatchability from set eggs (*p* < 0.05) and from fertile eggs (*p* < 0.01) increased, whereas embryo mortality decreased significantly (*p* < 0.01). Compared to the control, total lactic acid bacteria count increased and coliform bacteria count decreased significantly in boric acid‐supplemented groups (F300, W100 and W300) (*p* < 0.001). Egg production, feed conversion rate, viability, hatchability and intestinal bacterial load were similar among different feather colour varieties (*p* > 0.05). Feed consumption was highest in yellow and grey genotypes (*p* < 0.001).

**Conclusion:**

Boric acid can be used as a feed supplementation with positive effects on hatchability performance and intestinal health. Quail genotypes were generally similar in terms of the examined features.

## Graphical abstract

This study aimed to evaluate the effects of different dietary and water‐supplemented doses of boric acid on performance, hatchability, and intestinal bacterial load in Japanese quails of various feather colour genotypes. Boric acid supplementation improved hatchability rates and beneficially modulated intestinal microbiota without impairing production traits, supporting its use as a functional feed additive.

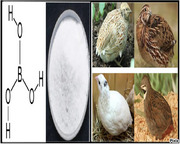

## 1 Introduction

Chickens are the most common poultry species used in human nutrition globally, but the farming of alternative poultry species has increased significantly in recent years. Quail is a poultry species characterized by low feed consumption and high egg production, and it does not require expensive equipment for breeding. Feather colour is considered a breed or line trait among quails. Studies have revealed that quails form 18 lines according to feather colour mutations (Baykalir et al. 2020; Eratalar and Okur 2022; Irmak, Denli, Kayri, Coşkun 2024).

Feed supplementations and nutritional supplements are becoming increasingly important in the poultry industry due to their wide range of beneficial effects, such as promoting growth and production, boosting immunity and protecting health (Abd El‐Hack et al. 2020). Antibiotics have been supplemented to feed and water since the 1940s to promote growth and therapeutic use in the poultry sector. However, serious problems have arisen in this regard, including the development of antimicrobial resistance, disruption of gut microbiota, accumulation of drug residues in poultry products and growing public health concerns related to food safety and environmental contamination. As a result, the use of antibiotics has been subject to restrictions and prohibitions (Yesilbag 2018). As the ban on antibiotics to improve performance in animal nutrition, the need for natural and residue‐free supplementations in animal agriculture has increased. For this purpose, researchers have intensively studied the effectiveness of prebiotics, probiotics, organic acids, essential oils, plant extracts and other supplementations because they stimulate growth and are not harmful to human health (Kutlu and Sahin 2017; Tufan et al. 2023; Irmak et al. 2024). Among these, boric acid stands out due to its promising biological properties (Apata 2009; Kheiri and Toghyani 2009; Eroglu et al. 2025).

Boric acid plays a vital role in various physiological processes, including lipid, mineral and energy metabolism, as well as supporting the immune, endocrine and nervous systems (Söğüt and Acar 2020; Sarı and Soysal 2021). It works by improving enzyme functions and maintaining cellular balance, which leads to enhanced nutrient utilization and overall health. When used as a dietary supplement in poultry, these effects contribute to increased egg weight, thicker eggshells, improved feed conversion and greater body weight gain (Ayasan et al. 2011; Pradhan et al. 2020). Kara (2022) found that the 20 mg/kg boric acid addition to the rations was sufficient for good eggshell quality and performance, and it was observed that higher doses had a negative effect on performance and shell quality. Jin et al. (2014) supplemented 0, 100, 200 and 400 mg/L boric acid to broiler water. They found that 200 and 400 mg/L doses negatively affected performance. The 100 mg/L dose significantly increased the immune organ index, whereas the 400 mg/L dose progressively worsened this index each week. Raimondi et al. (2006) reported that different derivatives of boric acid had bactericidal and fungicidal effects on *Staphylococcus aureus* ATCC 25923, *Escherichia coli* ATCC 25922, *Candida albicans* ATCC 10231 under in vitro conditions; Yılmaz (2012) reported that boric acid and sodium tetraborate had bactericidal and fungicidal effects on *S. aureus* ATCC 25923, *Acinetobacter* septics DSM 19415, *E. coli* ATCC 35218 and *Pseudomonas aeruginosa* ATCC 27853 under in vitro conditions.

This study hypothesized that different doses of boric acid supplemented to feed and water would influence egg performance, hatchability and intestinal bacterial load in Japanese quails with various feather colours and aimed to evaluate the potential effects of these treatments on productive and microbiological parameters in quails exhibiting different feather colour.

## 2 Materials and Methods

In this study, 300 Japanese quails (*Coturnix coturnix japonica*) with different feather colour genotypes—yellow (Y), white (W), grey (G) and black (B)—were used as the experimental subjects. At the beginning of the trial, the birds were 35 days old and had not yet started laying eggs. The experiment was conducted using a completely randomized 5 × 4 factorial design, incorporating five dietary treatments and four feather colour groups (yellow, white, grey and black). The quails were assigned to experimental groups with three replicates per treatment, each replicate consisting of a pen containing four females and one male from each feather colour. The birds were housed in laying quail cages comprising 15 pens arranged in 5 rows by 3 columns (Çimuka, BYK‐03‐5K). Environmental conditions were maintained at a temperature of 19–21°C with a 16‐h light and 8‐h dark photoperiod.

The dietary treatments included the following groups: control, receiving the basal diet (Table 1) without boric acid supplementation; F100, basal diet supplemented with 100 mg/kg boric acid; F300, basal diet supplemented with 300 mg/kg boric acid; W100, drinking water supplemented with 100 mg/L boric acid; and W300, drinking water supplemented with 300 mg/L boric acid. Each supplementation group consisted of 60 quails, with 15 birds from each feather colour (yellow, white, grey and black) group to ensure equal representation across treatments. The boric acid used (99.5% purity) had the following composition: boron trioxide (B_2_O_3_) > 56.0%, sulphate (SO_4_) < 0.02%, chlorite (Cl) < 0.001% and iron (Fe) < 0.0007%.

**TABLE 1:** Ingredients and nutrient composition of the basal diet.^a^

Ingredient	%	Nutritional composition	%
Corn	51.40	Dry matter	90.40
Wheat bran	9.00	Crude protein	18.00
Soya meal (44% HP)	22.00	Crude cellulose	4.40
Corn germ meal	2.00	Crude oil	5.35
Sunflower meal (45% HP)	4.30	Raw ash	10.19
Vegetable oil	3.50	Calcium^b^	2.50
Calcium phosphate	0.88	Phosphorus^b^	0.35
Calcium carbonate	4.50	Sodium^b^	0.18
Limestone	1.43	Lizin^b^	1.00
l‐Lysine hydrochloride	0.16	Methionine + cystine^b^	0.59
l‐Threonine	0.12	Treonin^b^	0.76
Sodium bicarbonate	0.16	Tryptophan^b^	0.25
Salt	0.20	ME, kcal/kg^b^	2800
Vitamin–mineral premix^c^	0.35		

^a^ In F100 and F300 groups, 100 and 300 ppm boric acid were added to the basal diets, respectively.

^b^ Calculated.

^c^ Vitamin‐mineral mix (per 1 kg): Vitamin A 15.500 IU; vitamin D3 3.500 IU, manganese 120 mg; zinc 100 mg; copper 16 mg; iron 40 mg; iodine 1.25 mg; selenium 0.30 mg; cobalt 200 mg.

Quails were supplemented with boric acid in their feed and water from 35 days old. To more accurately observe the effects of boric acid on and adaptation of quails to the environment, the data were taken after 15 days. The experimental process started when the quails were 50 days old and lasted 90 days (except the adaptation period). At the end of the experiment, a total of 40 quails, 8 quails (2 quails from each feather colour group) randomly selected from each group, were slaughtered for intestinal samples.

### 2.1 Performance Parameters

The number of eggs and feed consumption of quails were recorded every day. Feed consumption was defined as a daily food intake (g/quail/day) (Eroglu et al. 2024). Egg weight was defined as the average weight of each egg per pen (g/egg). The feed conversion ratio (g feed/g egg) was computed by dividing the quail's feed intake by the total weight of eggs they lay. (g feed/g egg). Egg production (%) was determined daily by dividing the number of eggs obtained by the number of quails on that day (Bayril and Eroğlu 2023). The amount of water given and left over each time was measured with a litre measuring tape and recorded. The water consumed was calculated according to the number of days and animals in the groups.

### 2.2 Incubation characteristics

Four hundred fifty eggs were collected in each replicate during the study. Eggs were stored for between 3 and 7 days, and then incubation was carried out. Incubation continued for 18 days, and at the end of the 18th day, unfertilized eggs and embryo deaths were determined. The following formulae were used to evaluate incubation results (Nowaczewski et al. 2010).

$$\begin{array}{ccc} \mathrm{Fertility}\mathrm{rate} & = & \left(\mathrm{Number}\mathrm{of}\mathrm{fertilized}\mathrm{eggs}/\right.\\ & & \left.\mathrm{Total}\mathrm{number}\mathrm{of}\mathrm{eggs}\mathrm{used}\right)\;\times \;100, \end{array}$$

$$\begin{array}{ccc} \mathrm{Hatching}\mathrm{from}\mathrm{set}\mathrm{eggs} & = & \left(\mathrm{Number}\mathrm{of}\mathrm{hatched}\mathrm{chicks}/\right.\\ & & \left.\mathrm{Total}\mathrm{number}\mathrm{of}\mathrm{eggs}\mathrm{used}\right)\;\times \;100, \end{array}$$

$$\begin{array}{ccc} \mathrm{Hatching}\mathrm{from}\mathrm{fertile}\mathrm{eggs}\; & = & \left(\mathrm{Number}\mathrm{of}\mathrm{hatched}\mathrm{chicks}/\right.\\ & & \left.\mathrm{Number}\mathrm{of}\mathrm{fertilized}\mathrm{eggs}\right)\;\times \;100, \end{array}$$

$$\begin{array}{ccc} \mathrm{Embryonic}\mathrm{death} & = & \left(\mathrm{Number}\mathrm{of}\mathrm{dead}\mathrm{embryos}/\right.\\ & & \left.\mathrm{Number}\mathrm{of}\mathrm{fertilized}\mathrm{eggs}\right)\;\times \;100. \end{array}$$

### 2.3 Gut Microbiota

After the cecum of the quails was detected, they were cut with a sterile scalpel with the help of forceps, then placed in sterile stomacher bags, and brought to the laboratory in a cold chain. Violet Red Bile Agar (VRB‐A) (Merck, Darmstadt/Germany) medium was used to determine the number of coliform bacteria. One gram of cecum content was taken into a sterile test tube, and 9 mL of sterile 0.1% peptone water was supplemented. In this way, 1/10 dilution was prepared. This preliminary dilution was used for the preparation of other decimal dilutions. Then, the plates were inoculated, and VRB‐A medium was supplemented. After the medium solidified, a second layer of VRB‐A medium was supplemented to the plates, and the plates were incubated at 35–37°C for 24 h. Colonies obtained at the end of incubation that were red, 1–2 mm in diameter, and had a precipitate zone around them were considered coliform group bacteria (FDA/BAM). After the samples taken from the cecum were inoculated onto the plates from the dilutions prepared as described above, De Man–Rogosa–Sharpe (MRS) agar (Merck, Darmstadt/Germany) medium was supplemented to the plates for lactic acid bacteria enumeration. The plates were incubated under anaerobic conditions at 37°C for 48 h. Cream‐coloured, spindle‐shaped (two pointed ends) colonies were considered lactic acid bacteria. Five randomly selected colonies were subjected to catalase and gram staining tests because lactic acid bacteria were catalase‐negative and gram‐positive.

### 2.4 Statistical Analyses

The research was carried out in a factorial trial design in randomized plots. After checking the homogeneity of variances (Levene's test) and normality of distributions (Shapiro–Wilk test), the GLM procedure was used to compare the groups using SPSS 21.0 (Chicago, IL). Tukey HSD test was applied for comparisons where significance was present. Data are presented in tables as mean ± standard error of the mean. Differences were considered significant when *p* ≤ 0.05. The following model was assumed in the analysis of all traits:

$$Y_{ijk}=\mu +a_{i}+b_{j}+{\left(a+b\right)}_{ij}+e_{j}$$

where *Y_ijk_* is the observed dependent variable; *μ* is the overall mean; *a_i_* is the effect of supplementation; *b_j_* is the effect of genotype (feather colour); (*a* + b)*_ij_* is the interaction of the supplementation with genotype; *e_ijk_* is the random error.

## 3 Results

### 3.1 Performance Parameters

The 50% and peak egg production age of the supplement and colour groups in the study are given in Table 2. In this study, 50% and peak production ages were insignificant between groups (*p* > 0.05). Boric acid supplementation in feed and water on feed intake values are shown in Table 3. Between the 1st and 15th days of the study, feed consumption was significantly lower in the F100 group (*p* ≤ 0.001). In colour groups, feed consumption values on Days 1–15, 16–30, 31–45, 46–60 and 1–90 differed (*p* ≤ 0.001). Feed consumption of white and black genotypes was lower than yellow and grey genotypes (*p* ≤ 0.001). Regarding the interactions between the groups, feed intake decreased in black and white‐coloured quails in the C group. In contrast, the highest feed intake was found in the yellow genotype in the F100 group, followed by grey, white and black genotypes, respectively (*p* ≤ 0.001). Similarly, in the F300, W100 and W300 groups, yellow and grey genotypes had high feed intake, whereas black and white genotypes had low feed intake (*p* ≤ 0.001).

**TABLE 2:** Peak and 50% egg production ages of the experimental group.

Groups	Age at 50% egg Production	Age at peak egg Production
**Boric acid supplementation**		
Control	48.00 ± 1.47	59.50 ± 3.22
F100	49.25 ± 1.43	56.25 ± 2.09
F300	48.00 ± 2.54	56.00 ± 3.74
W100	49.25 ± 2.52	56.75 ± 2.28
W300	50.50 ± 1.84	58.00 ± 3.55
***p* value**	**NS**	**NS**
**Feather colours**		
Yellow	52.00 ± 1.22	59.40 ± 2.04
White	47.40 ± 0.39	56.00 ± 2.28
Grey	52.00 ± 0.44	62.20 ± 1.74
Black	44.60 ± 1.16	51.60 ± 1.29
***p* value**	**NS**	**NS**

*Note*: Control: a basal diet; F100: basal diet + 100 mg/kg boric acid; F300: basal diet + 300 mg/kg boric acid; W100: 100 mg/L boric + drinking water; W300: 300 mg/L boric acid + drinking water.

Abbreviation: NS, not significant.

**TABLE 3:** Effects of boric acid supplemented to feed and water on feed consumption values (g).

Groups	1–15 days	16–30 days	31–45 days	46–60 days	61–75 days	76–90 days	1–90 days
**Boric acid supplementation**							
Control	25.57 ± 0.97^a^	28.54 ± 0.37	29.19 ± 0.17	29.70 ± 0.12	29.81 ± 0.11	29.58 ± 0.13	28.73 ± 0.28
F100	19.28 ± 1.39^b^	29.03 ± 0.26	28.76 ± 0.23	29.31 ± 0.24	29.74 ± 0.12	29.56 ± 0.18	27.61 ± 0.29
F300	26.71 ± 0.93^a^	29.45 ± 0.30	29.19 ± 0.37	29.72 ± 0.27	29.89 ± 0.26	29.76 ± 0.09	29.12 ± 0.30
W100	26.49 ± 1.03^a^	29.31 ± 0.36	29.28 ± 0.37	29.87 ± 0.17	29.89 ± 0.35	29.92 ± 0.34	29.13 ± 0.29
W300	25.97 ± 1.02^a^	28.88 ± 0.24	29.10 ± 0.20	29.73 ± 0.06	29.88 ± 0.07	29.67 ± 0.26	28.87 ± 0.26
*p* value	^***^	NS	NS	NS	NS	NS	NS
**Feather Colour**							
Yellow	28.58 ± 0.45^a^	29.81 ± 0.19^a^	29.74 ± 0.21^a^	29.91 ± 0.11^a^	29.95 ± 0.24	29.83 ± 0.06	29.64 ± 0.12^a^
White	22.25 ± 0.62^b^	28.21 ± 0.18^b^	28.47 ± 0.17^b^	29.20 ± 0.22^b^	29.66 ± 0.10	29.45 ± 0.25	27.84 ± 0.20^b^
Grey	27.41 ± 0.93^a^	29.73 ± 0.14^a^	29.66 ± 0.23^a^	30.06 ± 0.15^a^	29.98 ± 0.25	29.89 ± 0.20	29.45 ± 0.19^a^
Black	20.97 ± 1.19^b^	28.42 ± 0.30^b^	28.55 ± 0.18^b^	29.50 ± 0.07^ab^	29.79 ± 0.04	29.63 ± 0.21	27.81 ± 0.18^b^
*p* value	^***^	^***^	^***^	^***^	NS	NS	^***^
**Interaction (boric acid supplementation × feather colour)**							
*p* value	^***^	NS	NS	NS	NS	NS	NS

*Note*: Interaction: Boric acid supplementation feather colour, means within a same column not sharing a common superscript (a,b) are significantly different at *p* < 0.0, Control: a basal diet; F100: basal diet + 100 mg/kg boric acid F300: basal diet + 300 mg/kg boric acid; W100: 100 mg/L boric + drinking water; W300: 300 mg/L boric acid + drinking water.

Abbreviation: NS: not significant.

*** *p* < 0.001.

Egg production values are shown in Table 4, in the study, it was determined that boric acid supplemented to feed and water was not effective on egg production on Days 1–15, 16–30, 31–45, 46–60, 61–75, 76–90 and 1–90 (*p* > 0.05). Egg production was similar in colour groups except for Days 76–90 (*p* ≤ 0.05). In addition, it was determined that the interactions between the experimental groups had no significant effect on egg production (*p* > 0.05). Table 5 shows the effects of boric acid supplementation on egg weight. On Days 1–15, the lowest egg weight was found in the white genotype (*p* ≤ 0.05). Yellow, grey and black genotypes followed the white genotype. On Days 31–45, the lowest egg weight was determined in the white genotype, followed by the black, yellow and grey genotypes. Regarding the interactions between the experimental groups, it was found that egg weight was significantly lower in black‐coloured quails in the W100 group on the 61–75th day (*p* ≤ 0.05).

**TABLE 4:** Effects of boric acid supplemented to feed and water on egg production rates (%).

Groups	1–15 days	16–30 days	31–45 days	46–60 days	61–75 days	76–90 days	1–90 days
**Boric acid supplementation**							
Control	68.45 ± 5.81	86.65 ± 3.83	93.05 ± 1.11	90.32 ± 1.43	92.55 ± 1.75	92.55 ± 1.07	87.26 ± 1.78
F100	77.33 ± 4.39	86.06 ± 3.94	90.47 ± 2.67	90.47 ± 2.55	88.98 ± 3.52	85.86 ± 2.80	86.53 ± 2.40
F300	81.34 ± 2.37	89.08 ± 1.97	91.56 ± 2.25	83.92 ± 5.23	92.36 ± 1.49	89.88 ± 1.90	88.02 ± 1.41
W100	81.10 ± 5.24	84.72 ± 4.40	89.73 ± 2.24	90.32 ± 2.05	91.46 ± 2.08	87.05 ± 3.32	87.40 ± 2.18
W300	68.84 ± 3.46	85.96 ± 4.10	85.86 ± 3.72	89.33 ± 2.68	89.78 ± 4.76	85.61 ± 5.06	84.23 ± 3.33
*p* value	NS	NS	NS	NS	NS	NS	NS
**Feather colour**							
Yellow	84.16 ± 1.73	91.07 ± 1.31	92.38 ± 1.76	90.11 ± 1.56	92.85 ± 1.07	89.88 ± 1.46^ab^	90.07 ± 1.10
White	73.65 ± 5.80	90.15 ± 2.71	94.04 ± 1.40	87.22 ± 4.40	93.73 ± 2.56	92.69 ± 1.94^a^	88.58 ± 1.67
Grey	67.97 ± 4.05	84.04 ± 2.60	86.66 ± 2.39	88.33 ± 2.03	89.52 ± 2.33	89.16 ± 1.83^ab^	84.28 ± 1.93
Black	75.87 ± 4.41	80.71 ± 4.86	87.46 ± 2.88	89.84 ± 2.26	88.01 ± 3.74	81.03 ± 4.28^b^	83.82 ± 2.74
*p* value	NS	NS	NS	NS	NS	^*^	NS
**Interaction (boric acid supplementation × feather colour)**							
*p* value	NS	NS	NS	NS	NS	NS	NS

*Note*: Interaction: Boric acid supplementation × feather colour, means within a same column not sharing a common superscript (a,b) are significantly different at *p* < 0.05, Control: a basal diet; F100: basal diet + 100 mg/kg boric acid F300: basal diet + 300 mg/kg boric acid; W100: 100 mg/L boric + drinking water; W300: 300 mg/L boric acid + drinking water.

Abbreviation: NS: not significant.

* *p* ≤ 0.05.

**TABLE 5:** Effects of boric acid supplemented to feed and water on egg weight (g).

Groups	1–15 days	16–30 days	31–45 days	46–60 days	61–75 days	76–90 days	1–90 days
**Boric acid supplementation**							
Control	12.28 ± 0.18	12.45 ± 0.20	12.35 ± 0.20	12.49 ± 0.16	12.52 ± 0.18	12.42 ± 0.18	12.42 ± 0.17
F100	12.13 ± 0.18	12.19 ± 0.15	12.18 ± 0.15	12.04 ± 0.19	12.37 ± 0.16	12.31 ± 0.19	12.20 ± 0.16
F300	12.36 ± 0.12	12.34 ± 0.21	12.36 ± 0.17	12.14 ± 0.19	12.45 ± 0.21	12.52 ± 0.22	12.36 ± 0.17
W100	12.21 ± 0.13	12.42 ± 0.18	12.37 ± 0.18	12.49 ± 0.19	12.52 ± 0.27	12.18 ± 0.26	12.37 ± 0.18
W300	12.27 ± 0.17	12.35 ± 0.20	12.31 ± 0.22	12.59 ± 0.25	12.73 ± 0.25	12.51 ± 0.24	12.46 ± 0.20
*p* value	NS	NS	NS	NS	NS	NS	NS
**Feather colour**							
Yellow	12.26 ± 0.12^ab^	12.41 ± 0.17	12.45 ± 0.16^ab^	12.39 ± 0.17	12.63 ± 0.18	12.42 ± 0.19	12.43 ± 0.16
White	11.88 ± 0.14^b^	12.06 ± 0.14	11.93 ± 0.16^b^	11.94 ± 0.18	12.25 ± 0.19	12.15 ± 0.19	12.04 ± 0.15
Grey	12.42 ± 0.10^a^	12.58 ± 0.13	12.62 ± 0.18^a^	12.53 ± 0.15	12.72 ± 0.16	12.55 ± 0.14	12.57 ± 0.12
Black	12.43 ± 0.14^a^	12.34 ± 0.20	12.25 ± 0.16^ab^	12.53 ± 0.19	12.48 ± 0.21	12.43 ± 0.24	12.41 ± 0.16
*p* value	*	NS	^*^	NS	NS	NS	NS
**Interaction (boric acid supplementation × feather colour)**							
*p* value	NS	NS	NS	NS	NS	NS	NS

*Note*: Interaction boric acid supplementation × feather colour, means within a same column not sharing a common superscript (a,b) are significantly different at *p* < 0.05, Control: a basal diet; F100: basal diet + 100 mg/kg boric acid F300: basal diet + 300 mg/kg boric acid; W100: 100 mg/L boric + drinking water; W300: 300 mg/L boric acid + drinking water.

Abbreviation: NS: not significant.

* *p* ≤ 00.05.

The feed conversion ratio and viability rates are given in Table 6. In the supplementation groups, the best feed conversion ratio was determined in the F100 group on Days 1–15, and the worst value was determined in the control and W300 groups (*p* ≤ 0.05). The best feed conversion ratio was found in the black group (*p* ≤ 0.05).

**TABLE 6:** Effects of boric acid supplemented to feed and water on feed conversion efficiency (FCR) and viability (%).

Groups	1–15 days	16–30 days	31–45 days	46–60 days	61–75 days	76–90 days	1–90 days	Viability (1–90)
**Boric acid supplementation**								
Control	3.29 ± 0.28^a^	2.73 ± 0.17	2.54 ± 0.04	2.64 ± 0.03	2.58 ± 0.05	2.58 ± 0.04	2.66 ± 0.05	89.44 ± 4.67
F100	2.14 ± 0.20^b^	2.83 ± 0.14	2.63 ± 0.07	2.71 ± 0.07	2.76 ± 0.13	2.83 ± 0.08	2.63 ± 0.06	95.13 ± 3.32
F300	2.69 ± 0.15^ab^	2.70 ± 0.08	2.60 ± 0.08	3.24 ± 0.49	2.61 ± 0.05	2.67 ± 0.08	2.68 ± 0.06	92.77 ± 5.65
W100	2.95 ± 0.40^ab^	2.93 ± 0.26	2.65 ± 0.07	2.66 ± 0.06	2.63 ± 0.06	2.89 ± 0.16	2.71 ± 0.07	91.66 ± 5.98
W300	3.29 ± 0.28^a^	2.83 ± 0.21	2.82 ± 0.14	2.69 ± 0.12	2.78 ± 0.27	2.97 ± 0.30	2.81 ± 0.13	92.36 ± 5.75
*p* value	*	NS	NS	NS	NS	NS	NS	NS
**Feather colour**								
Yellow	2.78 ± 0.07^ab^	2.65 ± 0.05	2.60 ± 0.06	2.69 ± 0.05	2.56 ± 0.04	2.69 ± 0.06	2.65 ± 0.04	97.33 ± 1.81
White	2.93 ± 0.38^ab^	2.64 ± 0.11	2.54 ± 0.04	3.07 ± 0.39	2.63 ± 0.10	2.64 ± 0.07	2.62 ± 0.04	85.00 ± 6.31
Grey	3.40 ± 0.22 ^a^	2.85 ± 0.09	2.73 ± 0.06	2.73 ± 0.06	2.65 ± 0.06	2.69 ± 0.07	2.79 ± 0.05	97.33 ± 2.66
Black	2.38 ± 0.23 ^b^	3.08 ± 0.27	2.72 ± 0.12	2.65 ± 0.09	2.84 ± 0.21	3.13 ± 0.25	2.72 ± 0.11	89.44 ± 5.17
*p* value	^*^	NS	NS	NS	NS	NS	NS	NS
**Interaction (boric acid supplementation × feather colour)**								
*p* value	**NS**	**NS**	**NS**	**NS**	**NS**	**NS**	**NS**	**NS**

*Note*: Interaction: Group × colour, Means within a same column not sharing a common superscript (a,b) are significantly different at *p* < 0.05, Control: a basal diet; F100: basal diet + 100 mg/kg boric acid F300: basal diet + 300 mg/kg boric acid; W100: 100 mg/L boric + drinking water; W300: 300 mg/L boric acid + drinking water.

Abbreviations: B, black; G, grey; NS, not significant; W, white; Y, yellow.

* *p* ≤ 0.05.

**FIGURE 1:**
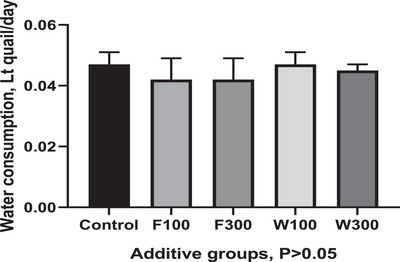
Boric acid supplementation affects water consumption in additive groups. Control: a basal diet without boric acid; F100: basal diet supplemented with 100 mg/kg boric acid F300: basal diet supplemented with 300 mg/kg boric acid; W100; 100 mg/L boric acid supplemented into drinking water; W300: 300 mg/L boric acid supplemented into drinking water. Column shows mean values of groups and bar shows the standard error of means.

The effects of boric acid supplementation to feed and water on water consumption in Japanese quails with different colour characteristics are given in Figure 1. It was determined that the research groups showed similar values in terms of water consumption (*p* > 0.05).

### 3.2 Hatching Characteristics

The data on hatching characteristics are shown in Table 7. There was a significant difference between the supplement groups (*p* ≤ 0.05). The highest hatching from fertile eggs was determined in the F300 group and the lowest in the control group (*p* ≤ 0.05). The hatching from set eggs was higher in the F300 group and lower in the control group (*p* ≤ 0.01). F100, W100 and W300 groups were similar to these groups (*p* > 0.05). Fertility rate values were similar among genotypes (*p* > 0.05). The embryo mortality rate was found that the control group was high and the F300 group was low (*p* ≤ 0.01). F100, W100 and W300 groups were similar to these groups (*p* > 0.05).

**TABLE 7:** Effects of boric acid supplemented to feed and water on incubation characteristics (%).

Groups	Hatching from set eggs	Hatching from fertile eggs	Fertility rate	Embryo Dead Rate
**Boric acid supplementation**				
Control	80.62 ± 4.59^b^	81.99 ± 4.63^b^	98.40 ± 0.91	18.00 ± 4.63^a^
F100	85.76 ± 2.84^ab^	92.06 ± 1.73^ab^	93.12 ± 2.44	7.93 ± 1.73^ab^
F300	93.68 ± 1.34^a^	96.17 ± 1.09^a^	97.43 ± 1.03	3.82 ± 1.09^b^
W100	86.73 ± 1.83^ab^	89.19 ± 2.06^ab^	97.36 ± 1.04	10.80 ± 2.06^ab^
W300	89.86 ± 2.01^ab^	92.24 ± 1.88^ab^	97.43 ± 0.97	7.75 ± 1.88^ab^
*p* value	^*^	**	**NS**	**
**Feather colour**				
Yellow	91.38 ± 1.31	93.78 ± 1.33	97.50 ± 0.89	6.21 ± 1.33
White	86.00 ± 1.83	90.03 ± 1.81	95.66 ± 1.53	9.96 ± 1.81
Grey	85.27 ± 3.01	89.04 ± 2.72	95.83 ± 1.77	10.95 ± 2.72
Black	86.66 ± 3.70	88.46 ± 3.70	98.00 ± 0.81	11.53 ± 3.70
*p* value	**NS**	**NS**	**NS**	**NS**
**Interaction (boric acid supplementation × feather colour)**				
*p* value	**NS**	**NS**	**NS**	**NS**

*Note*: Interaction boric acid supplementation × feather colour, means within a same column not sharing a common superscript (a,b) are significantly different at *p* < 0.05, Control: a basal diet; F100: basal diet + 100 mg/kg boric acid F300: basal diet + 300 mg/kg boric acid; W100: 100 mg/L boric + drinking water; W300: 300 mg/L boric acid + drinking water.

Abbreviation: NS: not significant.

* *p* ≤ 0.05.

### 3.3 Total Lactic Acid and Coliform Bacteria in the Intestinal Flora

Total lactic acid and coliform bacteria count in the intestinal flora are given in Table 8. Total lactic acid bacteria counts were higher in the F100, F300, W100 and W300 groups than the control group (*p* ≤ 0.001). Total lactic acid bacteria counts were significant in interactions F300 group and genotypes (*p* ≤ 0.05). In the F300 supplementation group, white and grey genotypes had higher bacterial counts, whereas yellow and black genotypes had lower counts (*p* ≤ 0.05). The highest total coliform bacteria count was found in the control group, followed by F100, F300, W100 and S300 groups (*p* ≤ 0.001).

**TABLE 8:** Effects of boric acid supplemented to feed and water on total lactic acid and coliform bacteria counts (log10 cfu/g).

Groups	Total lactic acid bacteria count	Total coliform acid bacteria count
**Boric acid supplementation**		
Control	4.06 ± 0.36^b^	6.11 ± 0.15^a^
F100	5.09 ± 0.21^a^	5.19 ± 0.18^ab^
F300	5.23 ± 0.20^a^	4.75 ± 0.30^b^
W100	5.28 ± 0.12^a^	3.78 ± 0.16^c^
W300	5.48 ± 0.21^a^	3.26 ± 0.27^c^
*p* value	^***^	^***^
**Feather colour**		
Yellow	4.59 ± 0.30	4.69 ± 0.43
White	5.25 ± 0.29	4.61 ± 0.43
Grey	5.16 ± 0.12	4.68 ± 0.35
Black	5.11 ± 0.25	4.48 ± 0.33
*p* value	NS	NS
**Interaction (boric acid supplementation × feather colour)**		
*p* value	*	NS

*Note*: Interaction: Boric acid supplementation × feather colour, means within a same column not sharing a common superscript (a,b) are significantly different at *p* < 0.05; Control: a basal diet; F100: basal diet + 100 mg/kg boric acid F300: basal diet + 300 mg/kg boric acid; W100: 100 mg/L boric + drinking water; W300: 300 mg/L boric acid + drinking water.

Abbreviation: NS: not significant.

* *p* ≤ 0.05.

*** *p* ≤ 0.001.

## 4 Discussion

### 4.1 Performance Parameters

In poultry, 50% and peak egg production age may vary with genotype and environmental effects. The lighting program, temperature and nutrition significantly affect 50% and peak egg production age (Tumova and Gous 2012). Ratriyantoa et al. (2018) found that the age at 50% and peak yield in quails were 58 and 84.40 days, respectively. In this study, 50% and peak yield ages of quails were 48.00 and 59.50 days, respectively.

In this study, boric acid supplementation to feed and water did not affect feed intake, egg production, egg weight, feed conversion ratio and viability. Similar to the findings of the present study, Sizmaz et al. (2021) found that egg weight and feed conversion values were similar between the study groups in laying hens in a study in which they supplemented boric acid (120 mg/kg), ascorbic acid (200 mg/kg) and their combination (120 mg/kg BA + 200 mg/kg AA) to the control (basal diet). The performance parameters mentioned above are closely related to energy metabolism in the organism. Although there is evidence that boric acid increases ATP production by participating in energy metabolism (Aysan et al. 2013), Ayasan et al. (2011) reported that dietary boric acid supplementation significantly affected the egg weight and feed conversion ratio of Japanese quails, whereas there was no significant difference between the control and boric acid supplemented groups regarding feed intake and egg production. In this study, it was found that feed intake was high in yellow and grey genotypes and low in white and black genotypes. The differences in feed intake values between the groups may be related to the quails’ genotypes. A study (Abdulla 2020) observed that quails with high carcass yield had high feed consumption. In the evaluation between the genotypes, although there were differences in egg production values in the periods examined, total egg production calculated on Days 1–90 was found to be similar between the genotypes. Egg weight was similar among the genotypes. The feed conversion ratio and survival rate were not statistically different between the colour groups.

This study found that boric acid supplementation did not increase or decrease water consumption. Water consumption was similar between the research groups. In this study, quails consumed 40–50 mL of water daily. Water consumption in poultry is generally calculated as 1.6–2.0 times more than the feed consumed, and it may vary with age and the amount and quality of feed dry matter content (El‐Saidy et al. 2015). Although there is no clear information about the effect of trace elements supplemented to the feed on the amount of water consumed daily, it is thought that the trace element may affect water consumption according to its hydroxy‐polymerization and acid solubility. As water is needed for such reactions, there may be a correlation between the intake of trace elements and water consumption (Bao et al. 2009). Jin et al. (2014) conducted a study to show the role of boric acid supplemented with drinking water at different doses on growth performance. They found that adding 100 mg boric acid/L to drinking water positively affected the growth performance; however, high‐dose boric acid addition (>200 mg/L) significantly inhibited the growth of broilers.

### 4.2 Hatching Characteristics

Boric acid supplemented to the feed at 300 ppm level decreased embryo mortality and increased hatchabilities compared to the control group. These findings indicate that boric acid has positive effects on hatching performance. When the control group was compared with the other additive groups, hatching from set and fertile eggs was the lowest. Similarly, the highest embryo mortality was also found in the control group. Sperm and oocyte quality are key factors directly impacting hatching performance. In quails, boric acid supplementation appears to enhance reproductive outcomes mainly through its antioxidative effects and regulation of vital metabolic pathways. By mitigating oxidative stress, boric acid safeguards spermatozoa and oocytes from damage caused by reactive oxygen species, thereby maintaining their cellular integrity and functional capacity (Simsek et al. 2024). In a study in which the effects of boric acid on fertility were examined, it was found that adding 40 ppm boric acid to the ration of bucks improved sperm quality (Krishnan et al. 2019). Although it was determined that boric acid did not affect egg production in chickens (El‐Saadany et al. 2017) and quails (Ayasan et al. 2011), it was reported to increase egg quality. In this study, the increase in the hatchability of the boric acid supplemented groups compared to the control group may be related to the improvement of sperm quality in male quails and egg quality in females. However, the effects of boric acid on fertility may vary depending on the dose. In a study conducted in rats, high doses of boric acid decreased sperm motility in males (Ku and Chapin 1994).

### 4.3 Total Lactic Acid and Coliform Bacteria in the Intestinal Flora

The intestinal microbiota describes the various types of microorganisms found in the intestines. The gut microbiota consists mainly of bacteria, viruses, yeasts, molds and other microorganisms. The microbiota has a significant impact on health. These effects are evaluated as protecting the organism against harmful pathogens, detoxifying and regulating the immune system (Stanley et al. 2014). In the study conducted in poultry, a low amount of lactobacilli and Clostridia and a high amount of anaerobic bacteria were determined in the microflora. The bacteria identified were anaerobic gram‐negative cocci, facultative anaerobic cocci and streptococci.

In this study, the lowest total lactic acid bacteria count was found in the control group among the supplementation groups. The highest coliform bacteria was found in the control group, followed by the F100, F300, W100 and W300 groups, respectively; this decrease in the total number of coliform bacteria can be considered an indicator that boric acid supplementation is significantly effective against coliform bacteria. In this context, boric acid supplementation can be regarded as a feed additive that supports the immune system through its antimicrobial properties. It exerts its effect by penetrating microbial cell membranes and disrupting both enzymatic and non‐enzymatic processes. Notably, boric acid has been shown to inhibit NADH oxidation, leading to reduced metabolic activity and ultimately causing microbial death (Hunt 1998). Additionally, it promotes the restoration of intestinal flora by increasing populations of lactic acid bacteria while suppressing coliform bacteria. Similarly, studies have been carried out in various animal species showing the antimicrobial activity of boric acid (Haesebrouck et al. 2009). Szałaj et al. (2018), boric acidic esters esterified with lipopeptidase showed a good antibacterial effect against *E. coli* by inhibiting *E. coli* type I signal peptidase (EcLepB) enzyme; Kivanc et al. (2018), hexagonal boric acid nitrite nanoparticles were found to be bactericidal against *Streptococcus mutans* 3. 3, *Staphylococcus pasteuri* M3, *Candida* sp. M25 and *S. mutans* ATTC 25175 prevented biofilm formation in the mouth by inhibiting the development of *S. mutans* 3.3, *S. mutans* ATTC 25175 and *Candida* sp. M25. In this study, boric acid is thought to similarly inhibit the coliform bacterial load in the intestine, creating a better environment for the growth of lactic acid bacteria.

## 5 Conclusion

According to the results of this study, it was determined that boric acid supplementation in feed and water at different doses had no significant positive or negative effect on egg production, feed intake and feed conversion. Boric acid supplementation significantly improved the hatching results, decreased coliform bacteria and increased lactic acid bacteria. It is thought that it can be used as a feed supplementation in poultry due to its positive effects on hatchability and intestinal health.

## Author Contributions

**Sultan Aslan** and **Ülkü Gülcihan Simsek**: conceptualization, experimental design, experiment performer, data analysis, drafted the manuscript, writing – review and editing. **Mehmet Eroğlu**: experiment performer, laboratory analysis, data analysis, writing – review and editing. **Seda İflazoğlu Mutlu**: experiment performer, laboratory analysis.

## Ethics Statement

All experimental procedures were approved by Elazığ Veterinary Control Institute Animal Experiments Local Ethics Committee (2018/05).

## Conflicts of Interest

The authors declare no conflicts of interest.

## Data Availability

The data presented in this study are available on request from the corresponding author.
